# Integration of the Gene Ontology into an object-oriented architecture

**DOI:** 10.1186/1471-2105-6-113

**Published:** 2005-05-10

**Authors:** Daniel Shegogue, W Jim Zheng

**Affiliations:** 1 Department of Biostatistics, Bioinformatics and Epidemiology, Medical University of South Carolina, 135 Cannon Street, Charleston, SC 29425 USA; 2 Bioinformatics Core Facility, Hollings Cancer Center, Medical University of South Carolina, 86 Jonathan Lucas St, Charleston, SC 29425 USA

## Abstract

**Background:**

To standardize gene product descriptions, a formal vocabulary defined as the Gene Ontology (GO) has been developed. GO terms have been categorized into biological processes, molecular functions, and cellular components. However, there is no single representation that integrates all the terms into one cohesive model. Furthermore, GO definitions have little information explaining the underlying architecture that forms these terms, such as the dynamic and static events occurring in a process. In contrast, object-oriented models have been developed to show dynamic and static events. A portion of the TGF-beta signaling pathway, which is involved in numerous cellular events including cancer, differentiation and development, was used to demonstrate the feasibility of integrating the Gene Ontology into an object-oriented model.

**Results:**

Using object-oriented models we have captured the static and dynamic events that occur during a representative GO process, "transforming growth factor-beta (TGF-beta) receptor complex assembly" (GO:0007181).

**Conclusion:**

We demonstrate that the utility of GO terms can be enhanced by object-oriented technology, and that the GO terms can be integrated into an object-oriented model by serving as a basis for the generation of object functions and attributes.

## Background

Complexity combined with an imprecise terminology has hindered the understanding of biology. A formal and structured vocabulary is now being developed to address this imprecise biology terminology. This vocabulary or Gene Ontology (GO) is being developed by the Gene Ontology Consortium (GOC) [1] to standardize the descriptions of gene products. Ontologies define the basic terms and relations comprising the vocabulary of a topic area, as well as the rules for combining terms and relations to define extensions to the vocabulary [2]. Despite these efforts, the mechanism of representing these terms lacks a unifying architecture that can be applied to the annotation of a gene product. However, computer science has developed a well-defined process and methodology for the development of software models. Adapting this process and methodology can orchestrate the assembly of biological models with integrated gene ontologies. In doing so, a standardized terminology and object-oriented model is created that can facilitate communication between biologists and computer scientists.

The Gene Ontology project is a collaborative effort that addresses the need for a controlled vocabulary that provides a consistent description of gene products in different databases [1]. The GO collaborators are developing three structured, controlled vocabularies that describe gene products, which have been classified into molecular function, biological process, and cellular component domains. GO terms are organized in structures called directed acyclic graphs (DAGs), which differ from hierarchies in that a 'child' (more specialized term) can have many 'parents' (less specialized terms). As part of these graphs, each component is given a GOid (unique identifier), and is associated with a GO definition. Collectively, these agreed upon terms are being developed to help explain various aspects of biology. When applied to a gene, that gene is annotated with a concise description of its molecular function, cellular location and associated biological processes. However, the GOC never intended to represent gene products or correlate ontological terms with these gene products [1]. To address this need, a Gene Ontology Annotation database [3] has been created to associate the GO terms with their gene product counterparts. With sustained effort, the descriptions of these gene products will ultimately be established. Still, much of the current bioinformatics work regarding GO has focused on constructing databases [4-7], applying it to other research areas [8-22], and building tools to mine the GO database. (For a description of some of these tools see [23].)

In addition, there has been an ongoing discussion regarding the depth of information obtained from the Gene Ontology [24]. It has been noted that there remains a need for a unifying architecture that integrates all three GO domains as part of a gene product's annotation. Furthermore, to enhance the Gene Ontology and facilitate its use as a cross-disciplinary tool, several additional issues need to be addressed. First, relationships between the biological processes, molecular functions and cellular components are not readily apparent [25-28]. Second, GO terms lack details. For instance, when one looks at molecular function there is no indication of what is inputted or outputted. Finally, existing tools such as GO-DEV [29] only contain software used for tool development and information retrieval, not software modeled directly after the three domains of the Gene Ontology. However, these issues can be resolved by integrating the Gene Ontology into an object-oriented system.

On a conceptual level, the Gene Ontology has features that support an object-oriented architecture. Consequently, the Gene Ontology can be applied and mapped to the fundamental concepts that form the object-oriented paradigm (i.e. class, object, inheritance, composition, polymorphism, and encapsulation) (Table 1). Furthermore, in an object-oriented sense, biological process terms are equivalent to high-level concepts. However, GO biological process terms do not contain descriptive information about the dynamics or static interactions defined by the terms. By translating a biological process into an object-oriented model the dynamic and static events occurring within a process can be represented. Building a static and dynamic model of a biological process requires defining the components of the process as well as the functions and attributes contained within these components. These components are biological entities (bioentities) that may include individual gene products, whose processes, functions and cellular components are captured in the Gene Ontology, or other higher-level entities such as gene product complexes.

The functions of gene products are the jobs or abilities that it has. In the GO terminology these are described in the molecular function domain. These are analogous to the operations that an object can perform in an object-oriented paradigm. Attributes, which define key properties of a component that when changed may alter the function of that component, may be defined by the cellular component and molecular function sections. For example, the cellular component domain can specify the place in a cell where a gene product is located. When there are multiple cellular components associated with a gene product, however, there is currently no mechanism to designate which cellular component represents the appropriate location.

The unified modeling language has been used to capture various aspects of biology [30-32]. These examples highlight the utility of the unified modeling language as a tool for biological data integration, and indicate that it can be applied to construct large, complex biological models. Therefore, to demonstrate the feasibility of integrating the Gene Ontology into an object-oriented model we have created unified modeling language (UML) representations of a GO biological process, "transforming growth factor beta (TGF-beta) receptor complex assembly" (GO:0007181).

The TGF-beta receptor pathway is involved in numerous cellular events including apoptosis, tumor development, differentiation, and development. These processes stem from the binding of TGF-beta to its cellular receptors. Briefly, dimerized TGF-beta 1 binds to TGF-beta receptor II (TβRII) and then TGF-beta receptor I (TβRI) complexes [33], causing their tetramerization (two type II receptors and two type I receptors) [34-36]. Constitutively activated type II receptor phosphorylates and activates type I receptor. Type I receptor propagates the signal by phosphorylating Smad 2, which is presented by the Smad Anchor for Receptor Activation (SARA) [37]. Phosphorylation of Smad destabilizes the Smad interaction with SARA, releasing it [38]. On TGF-beta stimulation, Smad 2 forms heterotrimeric complexes with Smad 4 and accumulates in the nucleus, binds DNA and remains for several hours [39-42]. Dephosphorylation allows Smad 2 to dissociate from Smad 4 and to be exported to the cytoplasm [43,44]. If the receptors are no longer active, then the Smads accumulate over time in the cytoplasm [44]. Alternatively, activated Smad 2 is ubiquitinated in the nucleus and undergoes proteasome-mediated degradation [45].

To create a unified model using the Gene Ontology we have taken the biological process term, "transforming growth factor beta (TGF-beta) receptor complex assembly" (GO:0007181), and used object-oriented models to define its dynamic and static architecture. We also show that one can augment the biological process domain terms by using the ontological terms and gene products associated with this process, and integrating them into an object-oriented model. Furthermore, we show that the molecular function, and cellular component domains can serve as a basis for the generation of object functions and attributes to create a standardized, comprehensive, and integrated model encompassing all the Gene Ontology domains.

## Results

### Converting GO directed acyclic graphs to object-oriented diagrams

**Figure 1:**
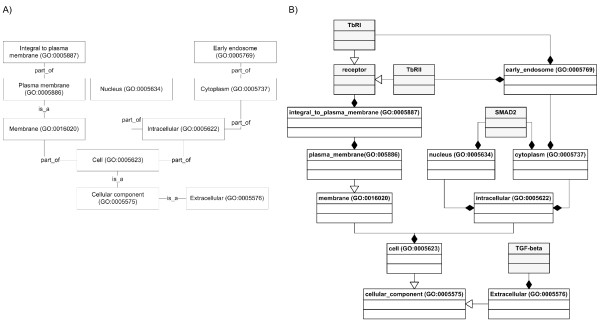
**The GO terms associated with the process, TGF-beta receptor complex assembly (GO: GO:0007181)**. Lines with solid diamonds (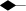) at the end indicate composition. These are read from the diamond end, for example, as 'cell' (GO:0005623) contains a 'membrane' (GO:0016020). Lines with open triangles (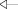) represent generalizations. These are read from the triangle end, as a 'membrane' (GO:0016020) is a general type of 'plasma membrane' (GO:0005886). **A) **Directed acyclic graph for the cellular component GO terms associated with TGF-beta receptor complex assembly (GO:0007181). **B) **Object-oriented representation of the DAG described in Figure 1A (white). Additional cellular components not represented by the current Gene Ontology, but essential to the TGF-beta receptor complex assembly process are shown in the gray boxes (i.e. TGF-beta, TGF-beta receptors, SMAD2)

The current DAG structure in which the Gene Ontology is represented is not readily amenable to transformation into software code. However, the architecture of directed acyclic graphs mimics that of an object-oriented class diagram. GO terms are presented in a parent-child hierarchy connected by 'is a' (generalizations) and 'part of' (composition) relationships. Read from top to bottom, the GO terms proceed from more specific to less specific. Directed acyclic graphs also allow the properties of multiple parent nodes to be inherited by child nodes, a form of multiple inheritance in object-oriented modeling. In figure 1A, cellular components related to the TGF-beta receptor complex are shown. One can create a UML diagram to mimic these relationships as shown in figure 1B. Since not all cellular components involved in the TGF-beta receptor complex assembly process are present in the current Gene Ontology, additional gene products based on literature searches were added to the object-oriented diagram (Figure 1B, shaded boxes). Relationships are captured as an object-oriented system through containment, composition and inheritance. Cellular components were decomposed into objects and connected via generalizations, which illustrate inheritance. Because these terms inherit all the attributes of their parents, only GO terms at the terminal nodes need be characterized in an object-oriented model. Relationships described as 'part of' were also extrapolated into object-oriented terms as composition.

The functions of gene products were also decomposed into object functions. The creation of object functions involved the transition from gene product functions to standardized GO molecular functions, and then to standardized, fully parameterized object functions. By applying formal ontological terms from the molecular function domain to gene products, object functions can be created with a consistent vocabulary. In table 2 we show the relationships between the function of a gene product defined in our model, and the GO molecular function term most closely corresponding to that cellular function. Here, we first compared ontology terms from the molecular function domain to those ascribed to individual gene products. Due to the incompleteness of the Gene Ontology, some gene product functions were extrapolated from the current literature, and then comparable GO molecular function terms were assigned to the gene products. Next, these molecular function terms were converted to object functions through reverse engineering. We identified the parameters that would normally be input into and output from a cellular reaction. In this way we defined the input and output parameters necessary for an object function. The object function itself was given the GOid that corresponds with its closest matching molecular function as defined by the GOid's definition. Together, object functions were created that are fully parameterized with inputs and outputs and that contain a standardized GO notation.

We conclude that it is feasible to create standardized functions for objects based on the current literature and an approved ontology. Together, ontological terms can be integrated into an object-oriented model paralleling the relationships, capturing the inherited aspects of the GO terminology, and providing a compact architecture while maintaining a standardized notation.

### Sequence diagram generation

**Figure 2:**
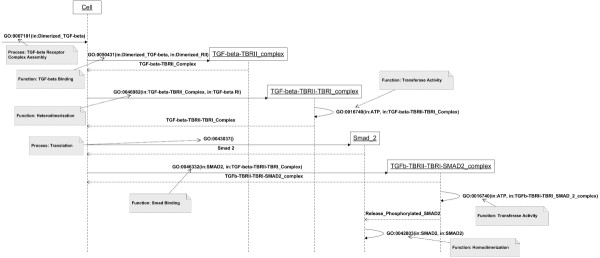
**An example of the sequence diagram showing the TGF-beta receptor complex assembly (GO:0007181)**. The binding of TGF-beta to its receptor (GO:0050431), receptor heterotetramerization (RI and RII homodimers, heterodimerizing)(GO:0046982), translation (GO:0043037), transferase activity (GO:0016740), and Smad 2 binding (GO:0046332) and activation (GO:0042301) are shown.

The GO biological process term, TGF-beta receptor complex assembly (GO:0007181), contains both static and dynamic features. The events of the TGF-beta receptor complex assembly (GO:0007181) process include TGF-beta binding (GO:0050431) to its receptors and SMAD binding (GO:0046332) and activation (GO:0042301). To capture the dynamic nature of these actions as an object-oriented software system, sequence diagrams were created. The events leading to Smad 2 activation are reflected chronologically in a high-level sequence diagram in Figure 2. The creation of the sequence diagram first entails identifying gene products and their functions by literature searches. Simple or complex bioentities are modeled as objects, which are represented by rectangles with vertical lifelines. Ontology terms taken from the molecular function domain that best corresponded to these functions were incorporated as object functions, which represent the functions of these gene products. These functions are implemented by the methods contained within the objects. Furthermore, these methods allow an object to communicate and interact with other objects, thus capturing cellular activities. To capture interactions between objects, one object can call a method of another object by connecting object lifelines in the sequence diagram (Figure 2). This invocation of a function of one object by another is described as one object sending a message to another object. Alternatively, a message may be passed from an object to itself as in the case of self-checks or autoactivation signals. In this way, real world processes may be captured using an object-oriented approach. For instance, to capture the formation of the TGF-beta and TGF-beta RII complex a GOid that closely corresponds to this ability is chosen as the method name. In this way the method can be cross-referenced to a GO term. Specifically, the method 'GO:0046982 (in Dimerized_TGF-beta, in Dimerized_RII)' references via the GOid, GO:0046982, "protein heterodimerization activity", and shows that a homodimer of TGF-beta and a homodimer of TGF-beta RII are needed to form the complex. Here, each dimer is thought of as a single entity, so the combination of these two entities is best represented as heterodimerization. A value of TGF-beta-TβRII_Complex is returned upon completion of the method as indicated by the return arrow. In contrast, the function call "GO:0042803 (in: SMAD2, in SMAD2)," references a self-call. The GOid can be cross-referenced to "protein homodimerization activity", which requires two SMAD2 components to generate the SMAD2 homodimer, but the message is passed only within the SMAD2 object. Furthermore, a message need not accept any parameters, as in the "translate()" function, which only returns a Boolean value indicating whether the action has occurred. Additional events such as TGF-beta RI activation, and Smad homodimerization, binding and activation are also reflected in figure 2. Together, this diagram demonstrates that the sequence of events occurring in the biological process, TGF-beta receptor complex assembly (GO:0007181), can be represented using the Gene Ontology, and can be integrated as part of the dynamics of an object-oriented software system.

### Activity diagram generation

**Figure 3:**
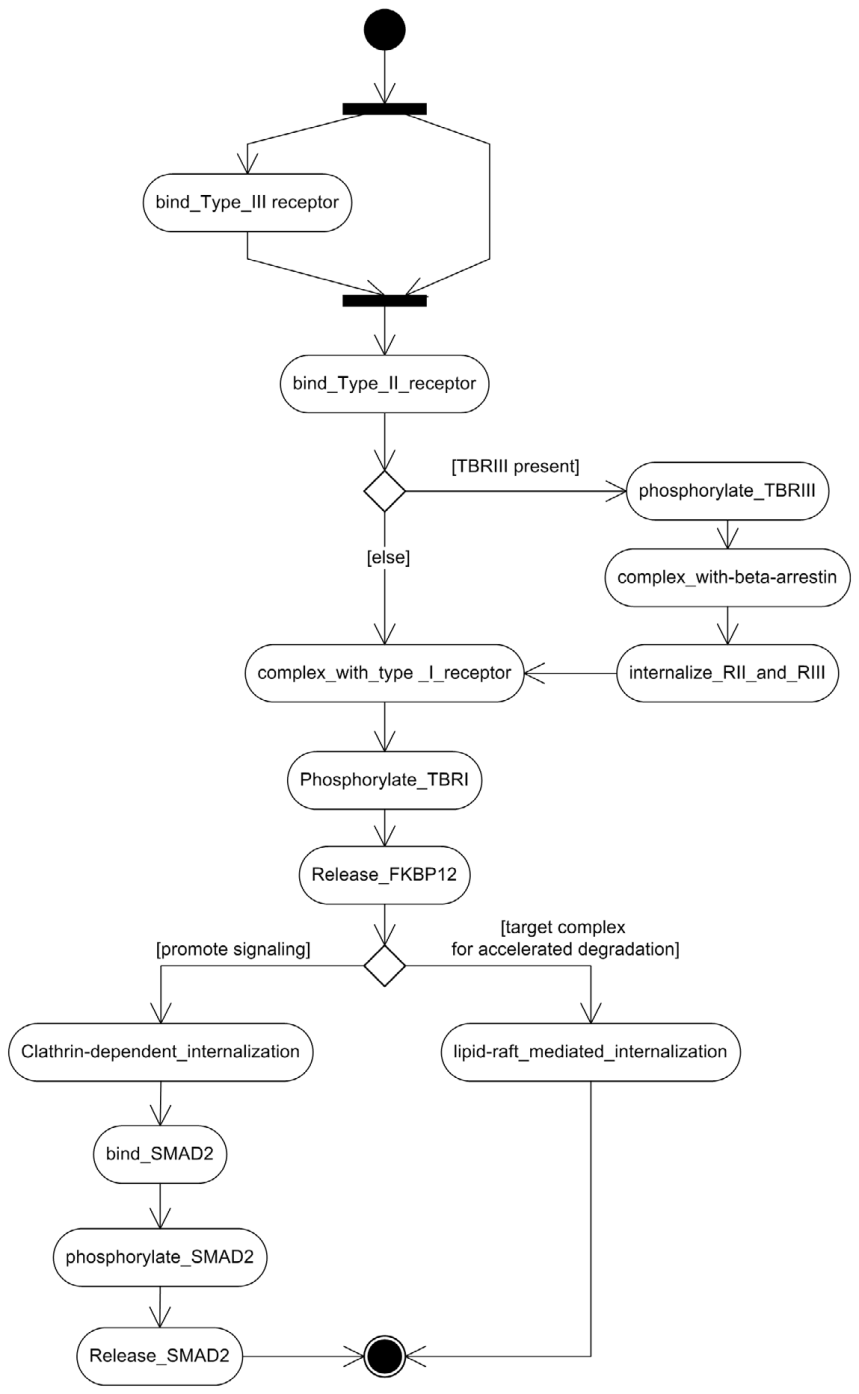
An example of an activity diagram showing the main and alternative flow of events occurring during TGF-beta receptor complex assembly (GO:0007181).

Biological processes are created from a series of complex events. While there may be one main event scenario that most frequently leads to a specific outcome often, alternative scenarios that lead to a process conclusion exist. This is exemplified by the sequence of events found in the TGF-beta receptor complex assembly (GO:0007181). For instance, TGF-beta may initially bind to TGF-beta RII or TGF-beta RIII. To capture these alternative events as part of the dynamic architecture, an activity diagram was created to reflect the initial stages of TGF-beta signaling (Figure 3). Unlike the sequence diagram, which captures main scenario events, the action sequence or flow of the activity diagram can portray alternative outcomes. Taking the example above, if TGF-beta binds to the type III receptor then an alternative flow of events occurs for a time that then returns to the main flow of events. Other possible divergences that were modeled included whether to internalize the TGF-beta receptors via clathrin-dependent or lipid raft-dependent mechanisms. These pathways lead to either complex degradation or signal promotion. Because complex degradation is not specified in our use case, for simplicity, this event is routed to the final state. However, the main success scenario, signal promotion, continues until SMAD2 is released and TGF-beta complex assembly is finished. Together, the dynamic events occurring during the biological process, TGF-beta receptor complex assembly (GO:0007181) are captured.

### Class diagram generation

**Figure 4:**
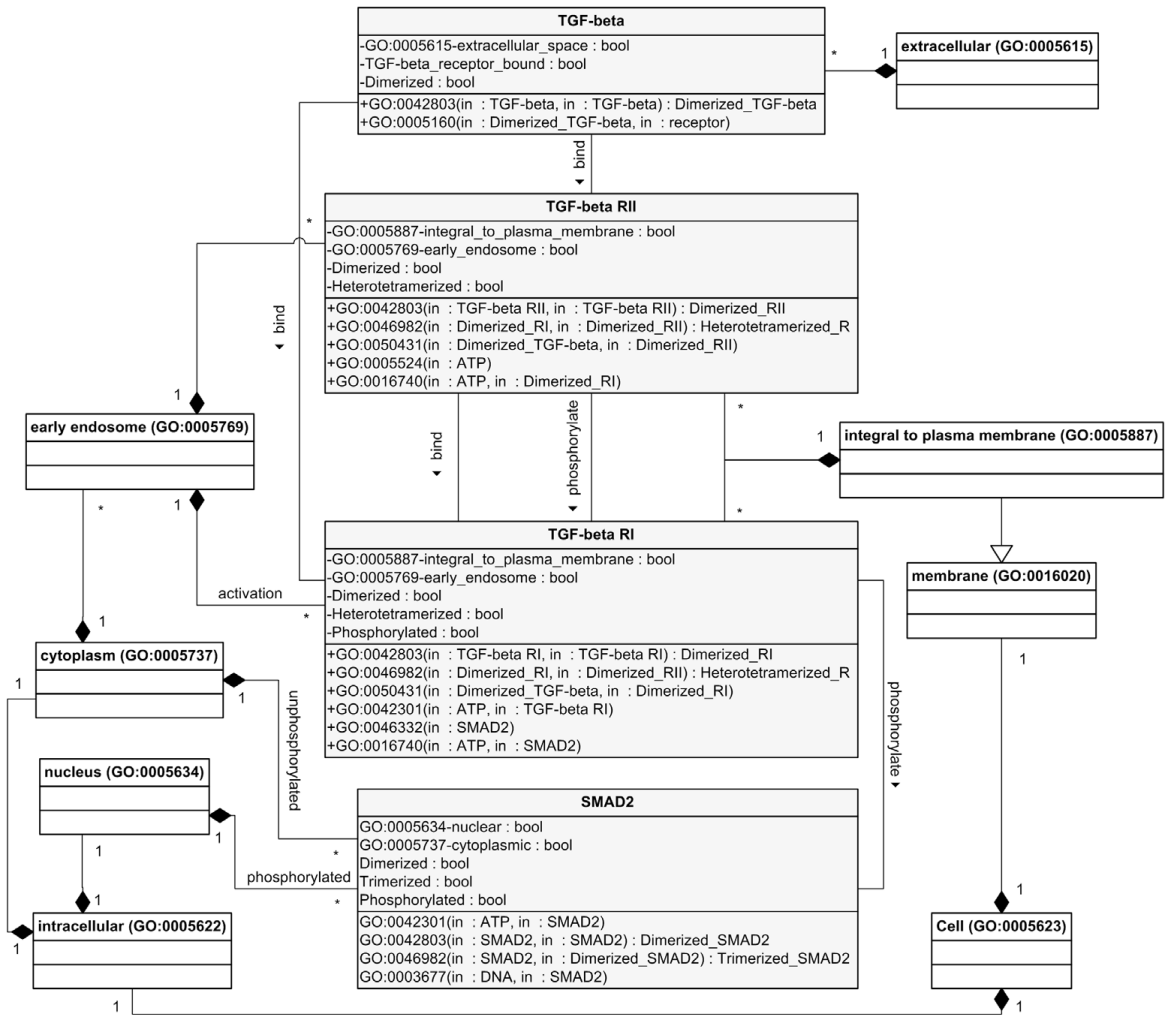
**An example of a class diagram showing the interactions between the components of the TGF-beta receptor complex (GO:0007181) (grayed)**. Cellular components containing these gene products are also shown. Due to space constraints functions are named using their corresponding GO ids. The class diagram was also constructed with a descriptive function name [see Additional file 8]. The GOid reference list is: GO:0042803, protein homodimerization activity; GO:0005160, TGF beta receptor binding; GO:0046982, protein heterodimerization activity, GO:0050431, TGF beta binding, GO:0005524, ATP binding; GO:0016740, transferase activity, GO:0046332, Smad binding; GO:0042301, phosphate binding; GO:0003677, DNA binding.

The major components of a biological system are bioentities with functions and interactions. Likewise, the center of an object-oriented software system is objects. Complex bioentities formed from multiple gene products along with their relationships, are contained within the biological system encompassing the biological process term, TGF-beta receptor complex assembly (GO:0007181). To represent the components that execute the process, we captured these components as bioentities with functions, and their interactions. The events of the TGF-beta receptor complex assembly (GO:0007181) process include TGF-beta binding (GO:0050431) to its receptors, and SMAD binding (GO:0046332) and activation (GO:0042301). To capture this static architecture, class diagrams were generated that model the bioentities, operations, and interrelationships that occur between TGF-beta, its receptors, and Smad 2. Similarly to figure 1, figure 4 captures the major components of the initial phases of TGF-beta signaling as objects with their associations, using an object-oriented representation. However, unlike figure 1, this object-oriented representation of the components of the main receptor complex is enhanced by the addition of attributes and functions. These objects were given attributes that describe important characteristics that if changed, might alter the function of a component. The functions of the objects, which parallel gene product functions, were generated from the sequence diagrams and were represented using Gene Ontology terms. These functions or operations are a declaration of the methods that an object may use. Together, the models generated using the described object-oriented methodology yield a software system representation of a biological process, TGF-beta receptor complex assembly, capturing both static and dynamic relationships annotated with Gene Ontology terms.

In addition, the UML notation provides a mechanism to specify inheritance that may be used to indicate an object that is the foundation for other objects. For instance, a TGF-beta receptor object might be a generalization of the TGF-beta receptor I object (data not shown). These specific objects inherit the properties of the receptor object. In addition, binary associations containing cardinalities may indicate the number of objects interacting with another. For instance, TGF-beta can interact with one to many receptors, while a receptor can only interact with one TGF-beta at a time (Fig. 4). Cellular compartments where these gene products can be found are also shown. Here, guard conditions are added to distinguish conditions under which each gene product might be found in a particular cellular compartment. In this way, a spatial representation of the TGF-beta receptor complex components is also achieved. These class diagrams demonstrate that the static structure of a biological system can be represented as an object-oriented model with integrated Gene Ontology terms. Collectively, the models generated using the described object-oriented methodology yield a software system representation of a biological system, capturing both static and dynamic relationships annotated with integrated Gene Ontology terms.

## Discussion

We have utilized the Gene Ontology to construct an object-oriented representation of the initial steps of TGF-beta signaling, and the gene products contained therein. In doing so, we have provided a standardized framework for the integration of Gene Ontology terms into gene product descriptions. By capturing all of the relevant GO terms in one model, the disjointed GO vocabulary is assembled into a cohesive structure. This cohesive structure encompasses the fundamental concepts of the object-oriented paradigm.

We proposed a solution to three unaddressed issues within the current Gene Ontology. First, while the Gene Ontology has helped to formalize the vocabulary that describes biological systems, it lacks a specific integration method. Currently, when applied to gene products, Gene Ontology terms are only categorically listed. Second, the Gene Ontology domains, biological process, molecular function and cellular component lack coherence. In particular, no association exists between domains. Finally, the current Gene Ontology defines GO terms, but gives no indication of what is necessary to accomplish a particular function, or process. To resolve these problems we defined an object-oriented methodology and architecture that provides a unifying framework to integrate all Gene Ontology domains.

The central dogma of the object-oriented paradigm revolves around several key aspects. Specifically, an object-oriented framework should accommodate the class, object, inheritance, composition, encapsulation and polymorphism concepts. As shown in table 1, gene products and other bioentities can be decomposed into objects, which are created based on template classes. These objects utilize inheritance to acquire the attributes and properties of more general objects. Complex classes can also be disassembled into subclasses using composition. Encapsulation allows the simplification of the model without sacrificing functionality. For instance, we do not need to know specific details regarding how a gene product is translated, just that a process that is encapsulated by the function 'translate()' can create a protein. However, if we wished to delve deeper into the mechanics of the translation process the layered architecture of the object-oriented system would allow us to do so. It is also worth noting that the modular nature of the object-oriented system closely resembles the recently discovered modular structure of biological networks [46-48]. This resemblance further indicates that biological systems can be easily modeled as object-oriented systems. Finally, polymorphism allows one to describe shared functions among different gene products. In this way, a function that may be shared broadly with other gene products can be uniquely specified for a particular gene product.

By applying object-oriented methodologies and concepts the various domains of the Gene Ontology can be coordinated into one model. Currently, the mechanisms in the biological process domain are veiled. There is no indication as to what gene products form the biological process, or what molecular functions are necessary to accomplish the process. Furthermore, the outcome of a specific process is not obvious. As in our example, a process such as TGF-beta receptor complex assembly (GO:0007181) does not give any indication of the components, dynamics or outcomes that occur during this process. However, by incorporating GO terms as attributes and functions we can discern relationships between the three domains. Likewise, the cellular components domain does not provide temporal or spatial clues when applied to gene products. For instance, GO terms 'extracellular' and 'intracellular' may both be associated with a particular gene product. However, the distinction between when a gene product is extracellular and when it is intracellular is not apparent. By applying object-oriented principles we can set extracellular and intracellular to Boolean values, and we can specify which location is the current (true) location of a gene product.

In addition, by using object-oriented principles a GO molecular function term can be augmented with parameters and outcomes. For example, the function "GO:0046982: protein heterodimerization activity" has different input and output parameters depending on the particular protein that contains the function. This type of polymorphic behavior, where one function can be performed in multiple ways is not supported by the Gene Ontology. For example, protein A may heterodimerize with protein B, whereas protein C heterodimerizes with protein D. From the Gene Ontology it is not readily apparent as to what is being inputted into the dimerization function. However, by applying an object-oriented architecture to function "GO:0046982: protein heterodimerization activity" we get "GO:0046982 (in: Protein A, in: Protein B): Protein AB". This format is an improvement to the unparameterized GO term in that the function can be cross-referenced to protein heterodimerization activity via its GO term, and we also see that for protein A to heterodimerize we need both protein A and protein B. In addition, we now observe that a new entity called protein AB is created from this function. By capturing the above details in an object-oriented model the GO term becomes far more useful for both biologists and computer scientists. Using an object-oriented approach the Gene Ontology domains are integrated into one cohesive model.

Integration of the Gene Ontology terms into an object-oriented representation offers several additional benefits. The object-oriented model provides additional levels of detail not found in the Gene Ontology. One of the strengths of object-oriented technology is the ability to capture the dynamics of a system. For example, sequence diagrams can chronologically order events in a biological process. Activity diagrams afford one the opportunity to envision different scenarios that might be occurring in a process. This additional level of detail significantly increases the depth of information that can be applied to the description of a biological process. State-transition diagrams also contribute to the realization of the full dynamics of a process by allowing the visualization of gene product states within a process. Furthermore, UML models can be translated into code, facilitating the creation of simulations.

The standardization of biological system modeling and integration is growing rapidly. A widely accepted example of the drive toward standardization is the Systems Biology Markup Language (SBML) [49], which has been adopted by more than 70 software tools [50]. The Gene Ontology is another example. However, each of the technologies, the Gene Ontology, the object-oriented approach, and SBML, has strengths and weaknesses. The Gene Ontology provides a standardized vocabulary but contains disconnected domains with no details regarding terms. SBML was developed to communicate biological models, with an emphasis on mathematical modeling of biological systems, but does not specify how to construct these models. Object-oriented technologies, on the other hand, provide a well-defined process for model creation and visualization, but have not been standardized for biology. However, the Gene Ontology, object-oriented paradigm, and SBML can form a new synergism when jointly applied to a common biological system model. These technologies are steps toward a unified approach to biological information integration, and studying biological phenomena at the systems level. Together, this unified approach will make biological system integration and analysis consistent, manageable and controllable, which is essential in handling complex systems, as demonstrated by decades of software industry experience.

While the described object-oriented approach can significantly enhance the annotation of gene products using the Gene Ontology, several challenges will need to be addressed. Specifically, object-orientation was not specifically designed for use in biological systems. Therefore, its use in capturing biological systems is not well defined. Furthermore, the Gene Ontology is still expanding and undergoing revisions. Consequently, in the near future it will still be necessary to do literature searches to define all the gene ontologies associated with a gene product. However, automated extraction of information for UML model generation and software implementation for simulations is under development, but is beyond the scope of this paper.

Future systems may also be implemented as software libraries in object-oriented programming languages (C++ and Java) for computer scientists to construct software for various applications and can be distributed as part of the GO-DEV toolkit for Gene Ontology development [29]. In addition, reformatting gene products with Gene Ontology terms will require the cooperation of multiple groups of biologists and computer scientists. However, we must take into consideration that a primary issue with this approach is the lack of people with cross-disciplinary skills able to comprehend both the biology and the computer science. Nonetheless, our own experience has shown that with supervision one biologist without a formal computer science background can learn to model a biological system using UML in a matter of months. Furthermore, automation of some of the annotation process will significantly reduce the human effort, but not eliminate the need for human annotators. Additional standards for automation will also need to be developed to thoroughly specify the process of object-oriented biological system integration. Despite these challenges the ultimate goal of creating a library of UML objects or modules integrated with Gene Ontology attributes and functions is worthwhile. Through this endeavor, biological processes could be assembled from these libraries for the development of simulation tools that will increase the productivity of biologists through increased insight into disease pathways and mechanisms.

## Conclusion

Here, we have demonstrated that Gene Ontology terms can be integrated into an object-oriented model. Furthermore, the object-oriented technology and methodologies used for this integration should improve the usability of these terms, and increase the depth of information that they contain. This work also serves as a framework for reverse-engineering biological gene products as objects in an object-oriented system. Together, this should facilitate additional collaborations between biologists and computer scientists.

## Methods

UML representations of the TGF-beta receptor complex assembly process were created following a software engineering process consisting of phases of requirement-gathering, analysis and design. UML models were generated using Microsoft Visio Pro. AmiGO [51] was used to determine Gene Ontology links for TGF-beta gene products.

### Requirement-gathering phase

#### Information collection

To define the requirements and collect the information necessary for the generation of the models, two approaches were necessary. First, annotations of the TGF-beta signaling pathways were conducted during an extensive literature review. Second, gene ontologies and Uniprot entries were searched to assign Gene Ontology terms to gene products. The attributes and the interactions of the TGF-beta signaling components were captured using class-responsibility collaboration (CRC) cards as described previously [52] [see Additional files 1, 2, 3, 4].

#### Use case development

Based on the gathered information, best-case and alternative scenarios were developed within a so-called "use case" to describe the TGF-beta receptor complex assembly process [see Additional file 5]. The use case also serves to define the boundary and scope of the TGF-beta model. For demonstration purposes the boundary of the system was limited to the steps TGF-beta receptor complex assembly. Therefore, alternative events such as receptor ubiquitination and degradation, as well as the specifics of SMAD 2 mobility were not captured in the dynamic models (i.e. sequence diagram).

### Analysis phase

#### Conceptual model generation

To provide an overview of the system and its interrelationships a conceptual based on the information defined in the requirement-gathering phase was generated [see Additional file 6]. This conceptual model integrated biological information, and represented TGF-beta and the cellular components involved in the complex assembly and their relationships in UML notation. By applying object-oriented analysis, the TGF-beta receptor complex assembly was decomposed into objects and component relationships were realized. However, information regarding component properties is hidden through encapsulation. This conceptual model defines the organization of the biological system and provides an overview of the components and their relationships.

### Design phase

#### State diagram generation

The dynamics of the system can also be captured using state diagrams, which can be used to describe the transitions and different states that a cellular component can exist [see Additional file 7]. In addition, multiple concurrent states can be illustrated using this UML notation.

#### Sequence, activity, class diagram generation

Sequence, activity and class diagrams have been used as an example to demonstrate the feasibility of generating an object-oriented representation of the biological process described by the GO term TGF-beta receptor complex assembly (GO:0007181), with Gene Ontology terms applied to generate these diagrams. Objects representing corresponding gene products are created, and their essential attributes are captured. Interactions among objects are also identified. For each interaction, a corresponding method is generated. This method is matched to a Gene Ontology term. The nature of the interaction determines the method parameters. The sequence of events is captured, and used to generate sequence diagrams. Scenarios are also generated for object interactions, and used to generate activity diagrams. The information captured in the sequence diagram and activity diagrams are used, along with the gene products attributes, to generate class diagrams.

## Authors' contributions

DS drafted the manuscript, constructed the models and participated in the design of the study. WJZ was the principal investigator, conceived of the project and guided its development. All authors read and approved the final manuscript.

## Supplementary Material

Additional File 1 **Class-responsibility-collaboration card for TGF-beta 1**. Attributes, collaborators and responsibilities of the specified protein are given. The attributes section allows the ordered listing of information not easily captured by the UML notation. The collaborator section lists the cellular components that interact with TGF-beta 1. The responsibilities section specifies the consequence of TGF-beta 1 interacting with its collaborator. This card allows TGF-beta 1 to be decomposed into an object containing attributes, operations and interactions. Click here for file

Additional File 2 **Class-responsibility-collaboration card for TGF-beta receptor II**. Attributes, collaborators and responsibilities of the specified protein are given. The attributes section allows the ordered listing of information not easily captured by the UML notation. The collaborator section lists the cellular components that interact with TGF-beta receptor II. The responsibilities section specifies the consequence of TGF-beta receptor II interacting with its collaborator. This card allows TGF-beta receptor II to be decomposed into an object containing attributes, operations and interactions. Click here for file

Additional File 3 **Class-responsibility-collaboration card for TGF-beta receptor I**. Attributes, collaborators and responsibilities of the specified protein are given. The attributes section allows the ordered listing of information not easily captured by the UML notation. The collaborator section lists the cellular components that interact with TGF-beta receptor I. The responsibilities section specifies the consequence of TGF-beta receptor I interacting with its collaborator. This card allows TGF-beta receptor I to be decomposed into an object containing attributes, operations and interactions. Click here for file

Additional File 4 **Class-responsibility-collaboration card for SMAD2**. Attributes, collaborators and responsibilities of the specified protein are given. The attributes section allows the ordered listing of information not easily captured by the UML notation. The collaborator section lists the cellular components that interact with SMAD2. The responsibilities section specifies the consequence of SMAD2 interacting with its collaborator. This card allows SMAD2 to be decomposed into an object containing attributes, operations and interactions. Click here for file

Additional File 5 **Use case describing the events leading to TGF-beta receptor complex assembly**. This use case defines the boundaries of the system model. Here, the main success and alternative scenarios leading to the assembly of the TGF-beta receptor complex are described. Click here for file

Additional File 6 **Conceptual diagram of the TGF-beta receptor complex, and the proteins that associate with this complex**. Gene products have been decomposed into objects. Object attributes and operations are hidden to reduce complexity. Gene products that comprise the receptor complex are shown in blue with their associated relationships in green. As the boundaries of the use case do not include them, other associated proteins that comprise the TGF-beta signaling pathway are not emphasized in further diagrams. Click here for file

Additional File 7 **State diagrams for the TGF-beta receptor complex components**. State diagrams describe the possible states in which a bioentity can exist. Concurrent states are separated by vertical dashed lines. **A) **State diagram for TGF-beta. **B) **State diagram for TGF-beta receptor II. **C) **State diagram for TGF-beta receptor I. **D) **State diagram for SMAD2. Click here for file

Additional File 8 **An example of a class diagram with expanded function names showing the interactions between the components of the TGF-beta receptor complex (GO:0007181) (grayed)**. Cellular components containing these gene products are also shown. GO function terms are shown with their corresponding GO ids. This format demonstrates a more user-friendly interface for reading the diagrams, whereas figure 4 is more suitable as a computer readable format. Click here for file

## Figures and Tables

**Table 1:** The use of object-oriented concepts in the integration of the Gene Ontology into an object-oriented model. Object-oriented terms, their definitions, and corresponding mechanisms of incorporating GO terms into an object-oriented model are shown. A specific example from the manuscript is also given. GO, Gene Ontology; DAG, directed acyclic graph; OOM, object-oriented model

**Object-Oriented Term**	Object-Oriented Definition *	**Object-Oriented use of the GO**	**Example**
Class	A class is a template from which object instances are created. It specifies the common characteristics that objects created from it will contain	Classes are created from gene products whose characteristics are defined by the GO molecular function and cellular component terms	The class Smad 2 is created based on the properties of the gene product Smad 2, which are defined by molecular functions such as "protein homodimerization' (GO:0042803) and 'ATP binding' (GO:0042301)
Object	An instance of a class that contains unique properties	Objects are created from the template classes, but may contain properties unique to a particular object	Two different Smad 2 objects may be created, one of which is phosphorylated, and one which is not
Inheritance	Relationships between classes, whereby a more specific class inherits all the properties and methods of the classes they belong to	Relationships defined by 'is a' are generalizations in which child classes of the DAG inherit the properties of the parent class (if a child class has multiple parent classes, multiple inheritance applies)	The cellular component 'plasma membrane' (GO:0005886) inherits the properties of the general class cellular component 'membrane' (GO:0016020)
Composition	Certain objects may be assembled from collections of other objects	'part_of' relationships defined in the GO DAG are rendered as composition relationships in an OOM	The 'membrane' (GO:0005623) and 'intracellular' (GO:0005622) space are part of the 'cell' (GO:0005623)
Polymorphism	The ability of an object to interpret messages differently when received by different objects	GO functions may change for different proteins and be given different input and output values	The function 'protein homodimerization activity' (GO:0042803) in the context of SMAD2 accepts two SMAD2s and outputs a dimerized SMAD2, whereas in the context of TGF-beta receptor II it accepts two receptors and outputs a dimerized receptor
Encapsulation	Hiding the state and implementation of an object	The exact mechanism by which an object is created is hidden in an OOM	The details involved in the translation (GO: 0043037) of Smad 2 are hidden, but a Smad 2 molecule is still created

*[53]

**Table 2:** The gene product functions described herein are listed with their associated GO molecular functions and parameters. These gene product functions are mapped to corresponding Gene Ontology molecular functions. These GO functions are integrated into an object-oriented model by amending them with input and output parameters, thereby creating object functions.

**Gene Product**	**Gene Product Function**	**Corresponding GO Term and GO ID**	**Input**	**Output**	**Figure Location**
TGF-beta	Dimerize	protein homodimerization activity (GO:0042803)	2X TGF-beta	Dimerized TGFβ	Fig.4
	bind TGF-beta receptor	TGF-beta receptor binding (GO:0005160)	TGFβ homodimer TGFβR homodimer	TGFβ-TGFβR complex	Fig.4

TβRII	Dimerize	protein homodimerization activity (GO:0042803)	2X TβRII	Dimerized RII	Fig.4
	TGF-beta binding	TGF-beta binding (GO:0060431)	TβRII homodimer TGFβ homodimer	TGFβ-TβRII heterotetramer	Fig.2, 4
	Heterotetramerize	protein heterodimerization activity (GO:0046982)	TβRI homodimer TβRII homodimer	TβRI-TβRII heterotetramer	Fig.2, 4
	phosphorylate RI	transferase activity (GO:0016740)	ATP TβRI homodimer	phosphorylated TβRI	Fig.2, 4

TβRI	Dimerize	protein homodimerization activity (GO:0042803)	2X TβRI	Dimerized RI	Fig.4
	Heterotetramerize	protein heterodimerization activity (GO:0046982)	TβRI homodimer TβRII homodimer	TβRI-TβRII heterodimer	Fig.2, 4
	TβRI activation	phosphate binding (GO:0042301)	ATP TβRI	phosphorylated TβRI	Fig.4
	bind SMAD2	Smad binding (GO:0046332)	SMAD2	TGFβ-TβRII-TβRI-SMAD2 complex	Fig.2, 4
	phosphorylate Smad	transferase activity (GO:0016740)	ATP SMAD2	phosphorylated SMAD2	Fig.2, 4

SMAD2	bind TβRI	TGF-beta receptor binding (GO:0005160)	TGFβ homodimer TβRI homodimer	TGFβ-TβRII-TβRI-SMAD2 complex	Fig.4
	SMAD2 activation	phosphate binding (GO:0042301)	ATP SMAD2	phosphorylated SMAD2	Fig.4
	Trimerize	protein heterodimerization activity (GO:0042803)	SMAD2 SMAD2 homodimer	Trimerized SMAD2	Fig.4
	activate transcription	DNA binding (GO:0003677)	DNA SMAD2	SMAD2-DNA complex	Fig.4
