# Supplementary material for: Integration of the Gene Ontology into an object-oriented architecture
Source: BMC Bioinformatics. 2005 May 10;6:113. doi: 10.1186/1471-2105-6-113 (PMC1156866; doi:10.1186/1471-2105-6-113)
Supplement: Additional File 1 — Class-responsibility-collaboration card for TGF-beta 1. Attributes, collaborators and responsibilities of the specified protein are given. The attributes section allows the ordered listing of information not easily captured by the UML notation. The collaborator section lists the cellular components that interact with TGF-beta 1. The responsibilities section specifies the consequence of TGF-beta 1 interacting with its collaborator. This card allows TGF-beta 1 to be decomposed into an object containing attributes, operations and interactions. [file 1471-2105-6-113-S1.pdf]

Class: **TGF-beta RII**	
Attributes:	
**Molecule Type:**	Protein
**Primary Sequence:**	
**Source:**	NCBI
**Accession Number:**	gi: 23308727
**Synonyms:**	
**Molecular Weight (kDa):**	70-80kD
**Cellular location**:	**GO:0005887:** integral to plasma membrane<br>**GO:0005768:** endosome
**Chromosome location:**	3p22
**Structure:**	
**Domain Information:**	
**Modifications:**	**GO:0005524:** ATP binding<br>**GO:0042803:** protein homodimerization activity (dimerize)
**Functions:**	**GO:0050431:** Transforming growth factor beta binding<br>**GO:0005160:** TGF-beta receptor binding<br>**GO:0016740:** transferase activity<br>**GO:0043130:** ubiquitin binding
**Processes involved in:**	**GO:0007179:** TGF-beta receptor signaling pathway<br>**GO:0007181:** TGF-beta receptor complex assembly<br>**GO:0007165:** Signal transduction<br>**GO:0006468:** protein amino acid phosphorylation

Responsibilities:	***Collaborators:***
Initiates formation of the receptor complex assembly, which leads to phosphorylation of RI by RII	TGF-beta (1)
Dimerization for receptor complex assembly	TGF-beta RII (1)
Phosphorylates type I receptor thereby propagating TGF-beta signal	TGF-beta RI (1)
TAB1 links the TGF-beta pathway to the MAPK pathway	TAB1(2)
Functional role unkown, but association results in phosphorylation of TRIP-1	TRIP-1 (3)

1) Massague, J., (1998) TGF-beta signal transduction, *Annu Rev Biochem*, **67(**753-91.
2) Shibuya, H., et al., (1996) TAB1: an activator of the TAK1 MAPKKK in TGF-beta signal transduction, *Science*, **272(5265),** 1179-82.
3) Chen, R.H., et al., (1995) A WD-domain protein that is associated with and phosphorylated by the type II TGF-beta receptor, *Nature*, **377(6549),** 548-52.
