# Supplementary material for: Integration of the Gene Ontology into an object-oriented architecture
Source: BMC Bioinformatics. 2005 May 10;6:113. doi: 10.1186/1471-2105-6-113 (PMC1156866; doi:10.1186/1471-2105-6-113)
Supplement: Additional File 2 — Class-responsibility-collaboration card for TGF-beta receptor II. Attributes, collaborators and responsibilities of the specified protein are given. The attributes section allows the ordered listing of information not easily captured by the UML notation. The collaborator section lists the cellular components that interact with TGF-beta receptor II. The responsibilities section specifies the consequence of TGF-beta receptor II interacting with its collaborator. This card allows TGF-beta receptor II to be decomposed into an object containing attributes, operations and interactions. [file 1471-2105-6-113-S2.pdf]

Class: **TGF-beta 1**	
Attributes:	
**Molecule Type:**	Protein
**Primary Sequence:**	
**Source:**	NCBI
**Accession Number:**	gi: 56605975
**Synonyms:**	
**Molecular Weight (kDa):**	44
**Cellular location**:	**GO:0005615:** extracellular space
**Chromosome location:**	19q13.1
**Structure:**	
**Domain Information:**	
**Modifications:**	**GO:0042803:** protein homodimerization activity (dimerize)
**Functions:**	**GO:0005160:** TGF-beta receptor binding
**Processes involved in:**	
	**GO:0007179:** TGF-beta receptor signaling pathway
	**GO:0007181:** TGF-beta receptor complex assembly
	**GO:0007165:** Signal transduction

Responsibilities:	***Collaborators:***
necessary for formation of signaling complex formation	TGF-beta RI (1)
necessary for formation of signaling complex formation	TGF-beta RII(1)
Dimerization possibly allows TGF-beta to bind one type I receptor and one type II receptor necessary for receptor complex assembly	TGF-beta (2)
clears growth factor from serum	alpha 2- macroglobulin receptor (3)
may change TGF-beta from latent to a bioactive conformation	Fibronectin (4)
may store bioactive TGF-beta in extracellular matrix	collagen IV (5)
interferes with binding of TGF-beta to receptors	60 kDa protein (6)

1) Massague, J., (1998) TGF-beta signal transduction, *Annu Rev Biochem*, **67(**753-91.
2) Sun, P.D. and Davies, D.R., (1995) The cystine-knot growth-factor superfamily, *Annu Rev Biophys Biomol Struct*, **24(**269-91.
3) LaMarre, J., et al., (1991) An alpha 2-macroglobulin receptor-dependent mechanism for the plasma clearance of transforming growth factor-beta 1 in mice, *J Clin Invest*, **87(1),** 39-44.
4) Fava, R.A. and McClure, D.B., (1987) Fibronectin-associated transforming growth factor, *J Cell Physiol*, **131(2),** 184-9.
5) Paralkar, V.M., Vukicevic, S., and Reddi, A.H., (1991) Transforming growth factor beta type 1 binds to collagen IV of basement membrane matrix: implications for development, *Dev Biol*, **143(2),** 303-8.
6) Piek, E., et al., (1997) Characterization of a 60-kDa cell surface-associated transforming growth factor-beta binding protein that can interfere with transforming growth factor-beta receptor binding, *J Cell Physiol*, **173(3),** 447-59.
