# Supplementary material for: Integration of the Gene Ontology into an object-oriented architecture
Source: BMC Bioinformatics. 2005 May 10;6:113. doi: 10.1186/1471-2105-6-113 (PMC1156866; doi:10.1186/1471-2105-6-113)
Supplement: Additional File 3 — Class-responsibility-collaboration card for TGF-beta receptor I. Attributes, collaborators and responsibilities of the specified protein are given. The attributes section allows the ordered listing of information not easily captured by the UML notation. The collaborator section lists the cellular components that interact with TGF-beta receptor I. The responsibilities section specifies the consequence of TGF-beta receptor I interacting with its collaborator. This card allows TGF-beta receptor I to be decomposed into an object containing attributes, operations and interactions. [file 1471-2105-6-113-S3.pdf]

Class: **TGF-beta RI**	
Attributes:	
**Molecule Type:**	Protein
**Primary Sequence:**	
**Source:**	NCBI
**Accession Number:**	gi: 4759225
**Synonyms:**	ALK-5, ACVRLK4
**Molecular Weight (kDa):**	53
**Cellular location**:	**GO:0005887:** integral to plasma membrane<br>**GO:0005768:** endosome
**Chromosome location:**	9q22
**Structure:**	
**Domain Information:**	
**Modifications:**	**GO:0005524:** ATP binding<br>**GO:0042803:** protein homodimerization activity (dimerize)
**Functions:**	**GO:0050431:** Transforming growth factor beta binding<br>**GO:0046332:** Smad binding<br>**GO:0016740:** transferase activity<br>**GO:0043130:** ubiquitin binding
**Processes involved in:**	**GO:0007179:** TGF-beta receptor signaling pathway<br>**GO:0007181:** TGF-beta receptor complex assembly<br>**GO:0007165:** Signal transduction<br>**GO:0006468:** protein amino acid phosphorylation

Responsibilities:	***Collaborators:***
Initiates formation of the receptor complex assembly, which leads to phosphorylation of RI by RII	TGF-beta (1)
Dimerization for receptor complex assembly	TGF-beta RI (1)
Binds TGF-beta RII, which results in RI activation	TGF-beta RII (1)
TGF-beta RI binds Smad 2 to phosphorylate it and propagate the TGF-beta signal	SMAD2 (2)
SARA recruits SMAD2 to the TGF-beta receptors facilitating SMAD2 phosphorylation	SARA (3)
interacts stably with the activated TGFbeta type I receptor, thereby blocking the association, phosphorylation, and activation of Smad2	SMAD7 (4)
Inhibits activation of TGF-beta receptor I by TGF-beta receptor II	FKBP12 (5)
Mediates proteasome-dependent degradation of activated TGF-beta receptor I via its E3 conjugating activity	Smurf1 (6)
Protein complex used for the degradation of TGF-beta receptor I to downregulate the TGF-beta signaling pathway	26S proteasome (6)

1) Massague, J., (1998) TGF-beta signal transduction, *Annu Rev Biochem*, **67(**753-91.
2) Macias-Silva, M., et al., (1996) MADR2 is a substrate of the TGFbeta receptor and its phosphorylation is required for nuclear accumulation and signaling, *Cell*, **87(7),** 1215-24.
3) Tsukazaki, T., et al., (1998) SARA, a FYVE domain protein that recruits Smad2 to the TGFbeta receptor, *Cell*, **95(6),** 779-91.
4) Hayashi, H., et al., (1997) The MAD-related protein Smad7 associates with the TGFbeta receptor and functions as an antagonist of TGFbeta signaling, *Cell*, **89(7),** 1165-73.
5) Wang, T., et al., (1996) The immunophilin FKBP12 functions as a common inhibitor of the TGF beta family type I receptors, *Cell*, **86(3),** 435-44.
6) Ebisawa, T., et al., (2001) Smurf1 interacts with transforming growth factor-beta type I receptor through Smad7 and induces receptor degradation, *J Biol Chem*, **276(16),** 12477-80.
