# Supplementary material for: Integration of the Gene Ontology into an object-oriented architecture
Source: BMC Bioinformatics. 2005 May 10;6:113. doi: 10.1186/1471-2105-6-113 (PMC1156866; doi:10.1186/1471-2105-6-113)
Supplement: Additional File 4 — Class-responsibility-collaboration card for SMAD2. Attributes, collaborators and responsibilities of the specified protein are given. The attributes section allows the ordered listing of information not easily captured by the UML notation. The collaborator section lists the cellular components that interact with SMAD2. The responsibilities section specifies the consequence of SMAD2 interacting with its collaborator. This card allows SMAD2 to be decomposed into an object containing attributes, operations and interactions. [file 1471-2105-6-113-S4.pdf]

Class: SMAD2	
Attributes:	
**Molecule Type:**	Protein
**Primary Sequence:**	
**Source:**	NCBI
**Accession Number:**	gi: 51173730
**Synonyms:**	MADH2, MADR2
**Molecular Weight (kDa):**	52
**Cellular location**:	**GO:0005737:** cytoplasm<br>**GO:0005634:** nucleus
**Chromosome location:**	18q21.1
**Structure:**	
**Domain Information:**	CDD:14787: MH1 domain;<br>MH1 is a small DNA binding domain, binding in an unusual way involving a beta hairpin structure binding to the major groove<br><br>CDD:14788: MH2 domain;<br>C terminal domain of SMAD family proteins, responsible for receptor interaction, transactivation, and homo- and heterooligomerization; also known as Domain B in dwarfin family proteins
**Modifications:**	**GO:0042301:** phosphate binding (phosphorylate)<br>**GO:0043130:** ubiquitin binding (ubiquitinated)<br>**GO:0042803:** protein homodimerization activity (dimerize)<br>**GO:0042803:** "trimerize"<br>**GO:0007183:** Smad protein heteromerization
**Processes involved in:**	**GO:0007179:** TGF-beta receptor signaling pathway<br>**GO:0007181:** TGF-beta receptor complex assembly<br>**GO:0043037:** Translation<br>**GO:0005160:** TGF-beta receptor binding<br>**GO:0030579:** ubiquitin-dependent Smad protein catabolism<br>**GO:0007184:** Smad protein nuclear translocation<br>**GO:0008134:** transcription factor binding

Responsibilities:	***Collaborators:***
SMAD2 propagates the TGF-beta signal via its phosphorylation by TGF-beta RI, which activates the SMAD2 protein and promotes gene expression	TGF-beta RI (Wu et al., 2001)
Trimerization for SMAD2 binding and nuclear translocation	SMAD2 (Kawabata et al., 1998)
Heterodimerization for SMAD4 binding and nuclear translocation	SMAD3 (Nakao et al., 1997)
Smad2 binds to SMAD4 facilitating Smad4 translocation to the nucleus where it binds to DNA	SMAD4 (Souchelnytskyi et al., 1997)
SARA recruits SMAD2 to the TGF-beta receptors facilitating SMAD2 phosphorylation	SARA (Tsukazaki et al., 1998, Wu et al., 2000)
E2 conjugating enzyme begins ubiquitination process for degradation of SMAD2	UbcH5b/c (Lin et al., 2000, Zhang et al., 2001)
Mediates proteasome-dependent degradation of activated SMAD2 via its E3 conjugating activity	Smurf2 (Lin et al., 2000)
Protein complex used for the degradation of SMAD2 to downregulate the TGF-beta signaling pathway	26S proteasome (Lo and Massague, 1999)

Kawabata, M., Inoue, H., Hanyu, A., Imamura, T., and Miyazono, K., (1998) Smad proteins exist as monomers in vivo and undergo homo- and hetero-oligomerization upon activation by serine/threonine kinase receptors, *Embo J*, **17,** 4056-4065.

Lin, X., Liang, M., and Feng, X.H., (2000) Smurf2 is a ubiquitin E3 ligase mediating proteasome-dependent degradation of Smad2 in transforming growth factor-beta signaling, *J Biol Chem*, **275,** 36818-36822.

Lo, R.S. and Massague, J., (1999) Ubiquitin-dependent degradation of TGF-beta-activated smad2, *Nat Cell Biol*, **1,** 472-478.

Nakao, A., Imamura, T., Souchelnytskyi, S., Kawabata, M., Ishisaki, A., Oeda, E., Tamaki, K., Hanai, J., Heldin, C.H., Miyazono, K., and ten Dijke, P., (1997) TGF-beta receptor-mediated signalling through Smad2, Smad3 and Smad4, *Embo J*, **16,** 5353-5362.

Souchelnytskyi, S., Tamaki, K., Engstrom, U., Wernstedt, C., ten Dijke, P., and Heldin, C.H., (1997) Phosphorylation of Ser465 and Ser467 in the C terminus of Smad2 mediates interaction with Smad4 and is required for transforming growth factor-beta signaling, *J Biol Chem*, **272,** 28107-28115.

Tsukazaki, T., Chiang, T.A., Davison, A.F., Attisano, L., and Wrana, J.L., (1998) SARA, a FYVE domain protein that recruits Smad2 to the TGFbeta receptor, *Cell*, **95,** 779-791.

Wu, G., Chen, Y.G., Ozdamar, B., Gyuricza, C.A., Chong, P.A., Wrana, J.L., Massague, J., and Shi, Y., (2000) Structural basis of Smad2 recognition by the Smad anchor for receptor activation, *Science*, **287,** 92-97.

Wu, J.W., Hu, M., Chai, J., Seoane, J., Huse, M., Li, C., Rigotti, D.J., Kyin, S., Muir, T.W., Fairman, R., Massague, J., and Shi, Y., (2001) Crystal structure of a phosphorylated Smad2. Recognition of phosphoserine by the MH2 domain and insights on Smad function in TGF-beta signaling, *Mol Cell*, **8,** 1277-1289.

Zhang, Y., Chang, C., Gehling, D.J., Hemmati-Brivanlou, A., and Derynck, R., (2001) Regulation of Smad degradation and activity by Smurf2, an E3 ubiquitin ligase, *Proc Natl Acad Sci U S A*, **98,** 974-979.
