# Supplementary material for: Integration of the Gene Ontology into an object-oriented architecture
Source: BMC Bioinformatics. 2005 May 10;6:113. doi: 10.1186/1471-2105-6-113 (PMC1156866; doi:10.1186/1471-2105-6-113)
Supplement: Additional File 5 — Use case describing the events leading to TGF-beta receptor complex assembly. This use case defines the boundaries of the system model. Here, the main success and alternative scenarios leading to the assembly of the TGF-beta receptor complex are described. [file 1471-2105-6-113-S5.pdf]

Primary Actor: **TGF-beta**
Pre-conditions: TGF-beta and receptors must be present.
Post-conditions: SMAD 2 is phosphorylated and released from TGF-beta receptor I.

Main Success Scenario (or Basic Flow):

1. TGF-beta homodimerizes (GO:0042803)
2. TGF-beta binds TbRI-TbRII complexes (1) (GO:0050431)
3. Type I and II receptors tetramerize (2 type II receptors and 2 type I receptors) (2-4) (GO:0046982)
4. Constitutively activated Type II receptor phosphorylates and activates type I receptor (GO:0016740, GO:0042301)
5. TGF-beta and receptors are internalized
6. Type I receptor propagates signal by phosphorylating Smad 2 (GO:0046332, GO:0016740, GO:0042301)
7. The phosphorylation of Smad2 and formation of the Smad complex results in the release of activated Smads from TGF beta RI

Extensions (or Alternative Flows):

2a. Dimerized TGF-beta 1 binds to homodimeric TGF-beta receptor III (GO:0042803, GO:0046982)
  1. TGF-beta receptor III presents TGF-beta to Type II receptor and Type I receptor (1)
  2. TGF-beta receptor III can be phosphorylated by TGF beta RII (GO:0016740, GO:0042301)
    a. TGF-beta receptor III interacts with beta-arrestin, which mediates endocytosis of TGF-beta receptor III and TGF-beta RII (5)

3a. In an allosteric model, binding of ligand to the type II receptor is required to induce a conformational change in the ligand, which leads to exposure of the binding epitope of the type I receptor (6)
3b. In a cooperative model, the ectodomain of the type I receptor interacts with an extended surface that relies on the formation of the type II receptor-ligand complex (6)
4a. FKBP12 inhibits TGF-beta signaling by binding to the unphosphorylated type I receptor (7)
5a. Internalization via lipid raft-caveolar compartments containing receptor bound to Smad7-Smurf2
  1. results in accelerated receptor turnover by promoting poly-ubiquitination (8)

5b. Clathrin-dependent TGF-beta receptor internalization
  1. activated TGF-beta receptor complex is internalized via coated vesicles to SARA-containing early endosomes promoting Smad signaling (8)

1) Rodriguez, C., et al., (1995) Cooperative binding of transforming growth factor (TGF)-beta 2 to the types I and II TGF-beta receptors, *J Biol Chem*, **270(27),** 15919-22.
2) Brown, C.B., et al., (1999) Requirement of type III TGF-beta receptor for endocardial cell transformation in the heart, *Science*, **283(5410),** 2080-2.
3) Massague, J., (1998) TGF-beta signal transduction, *Annu Rev Biochem*, **67(**753-91.
4) Yamashita, H., et al., (1994) Formation of hetero-oligomeric complexes of type I and type II receptors for transforming growth factor-beta, *J Biol Chem*, **269(31),** 20172-8.
5) Chen, W., et al., (2003) Beta-arrestin 2 mediates endocytosis of type III TGF-beta receptor and down-regulation of its signaling, *Science*, **301(5638),** 1394-7.
6) Hart, P.J., et al., (2002) Crystal structure of the human TbetaR2 ectodomain--TGF-beta3 complex, *Nat Struct Biol*, **9(3),** 203-8.
7) Huse, M., et al., (1999) Crystal structure of the cytoplasmic domain of the type I TGF beta receptor in complex with FKBP12, *Cell*, **96(3),** 425-36.

8) Di Guglielmo, G.M., et al., (2003) Distinct endocytic pathways regulate TGF-beta receptor signalling and turnover, *Nat Cell Biol*, **5(5),** 410-21.
