# Supplementary figures and images for: Integration of the Gene Ontology into an object-oriented architecture
Source: BMC Bioinformatics. 2005 May 10;6:113. doi: 10.1186/1471-2105-6-113 (PMC1156866; doi:10.1186/1471-2105-6-113)

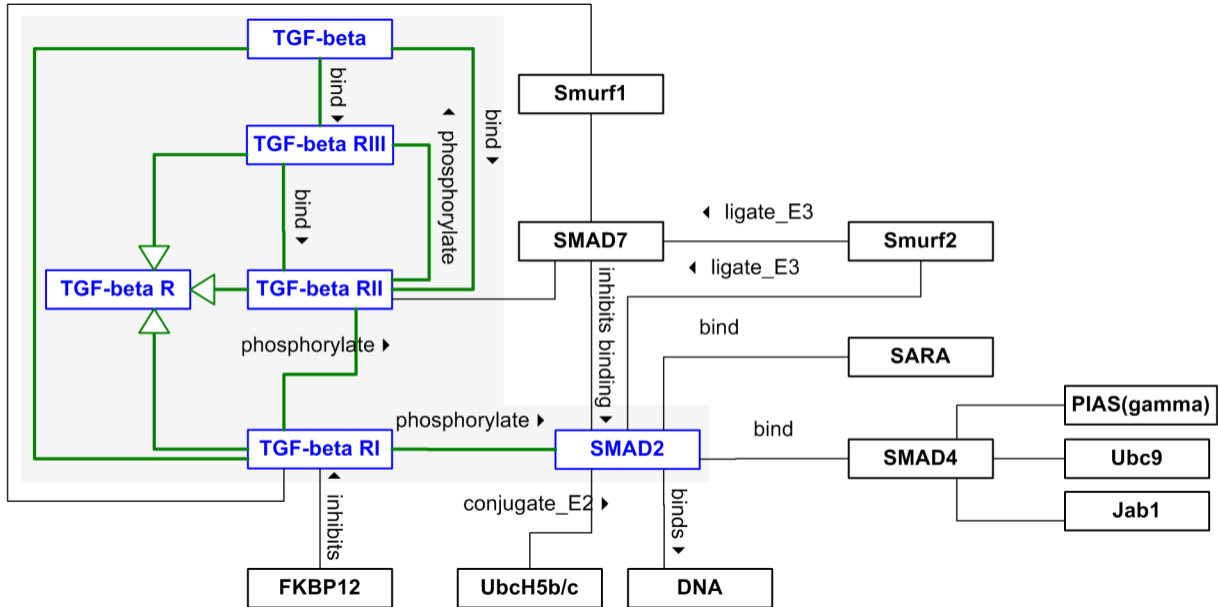

Supplement: Additional File 6 — Conceptual diagram of the TGF-beta receptor complex, and the proteins that associate with this complex. Gene products have been decomposed into objects. Object attributes and operations are hidden to reduce complexity. Gene products that comprise the receptor complex are shown in blue with their associated relationships in green. As the boundaries of the use case do not include them, other associated proteins that comprise the TGF-beta signaling pathway are not emphasized in further diagrams. [file 1471-2105-6-113-S6.pdf]

A) TGF-beta

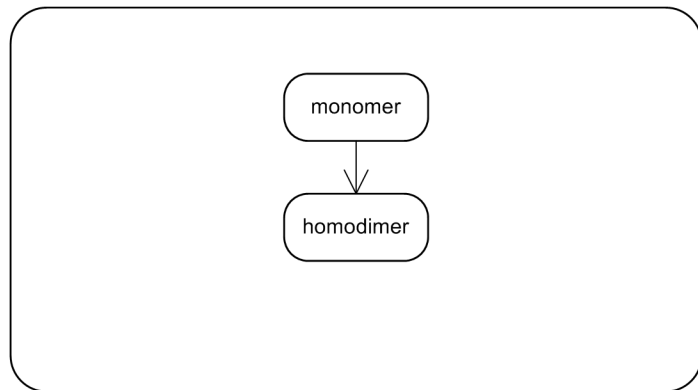

B) TBR1

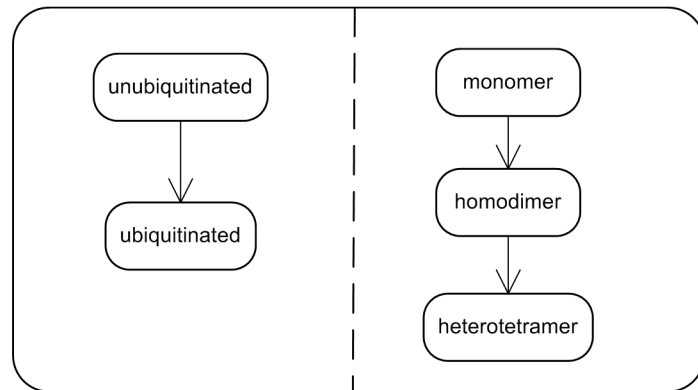

C) TBRI

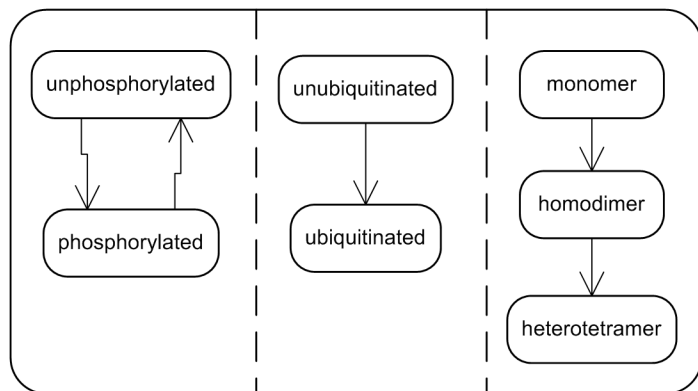

D) SMAD2

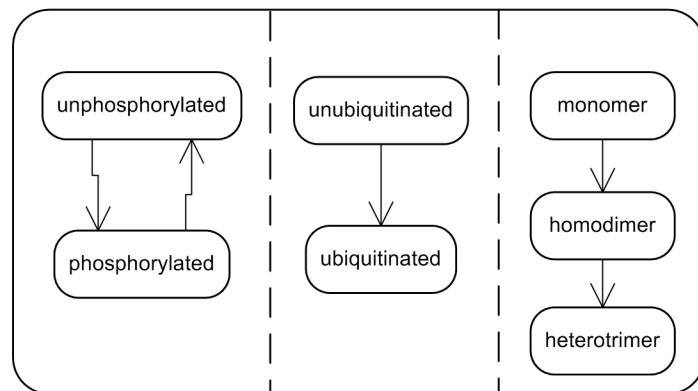

Supplement: Additional File 7 — State diagrams for the TGF-beta receptor complex components. State diagrams describe the possible states in which a bioentity can exist. Concurrent states are separated by vertical dashed lines. A) State diagram for TGF-beta. B) State diagram for TGF-beta receptor II. C) State diagram for TGF-beta receptor I. D) State diagram for SMAD2. [file 1471-2105-6-113-S7.pdf]

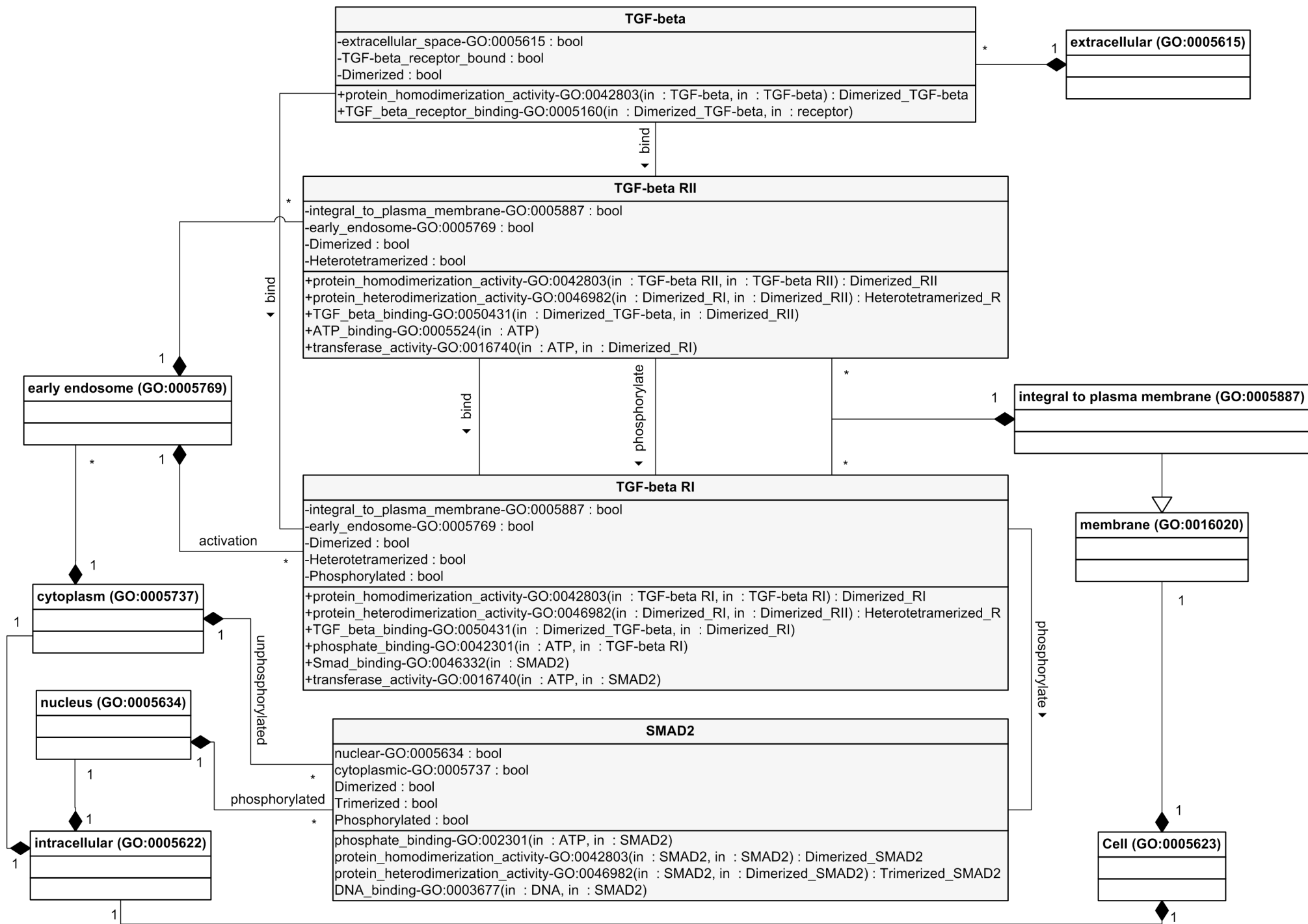

Supplement: Additional File 8 — An example of a class diagram with expanded function names showing the interactions between the components of the TGF-beta receptor complex (GO:0007181) (grayed). Cellular components containing these gene products are also shown. GO function terms are shown with their corresponding GO ids. This format demonstrates a more user-friendly interface for reading the diagrams, whereas figure 4 is more suitable as a computer readable format. [file 1471-2105-6-113-S8.pdf]
